# The Effect of Induced Regulatory Focus on Frontal Cortical Activity

**DOI:** 10.3390/bs14040292

**Published:** 2024-04-01

**Authors:** Yiqin Lin, Xiaomin Sun

**Affiliations:** Beijing Key Laboratory of Applied Experimental Psychology, National Demonstration Center for Experimental Psychology Education, Faculty of Psychology, Beijing Normal University, Beijing 100875, China; 202031061050@mail.bnu.edu.cn

**Keywords:** regulatory focus, frontal cortical activity, approach and avoidance motivation

## Abstract

The motivation–direction model has served as the primary framework for understanding frontal cortical activity. However, research on the link between approach/avoidance motivation and left/right frontal cortical activity has produced inconsistent findings. Recent studies suggest that regulatory systems may offer a more accurate explanation than the motivational direction model. Despite being regulatory systems, the relationship between regulatory focus and frontal cortical activity has received limited attention. Only one experimental study has explored this connection through correlational analysis, yet it lacks causal evidence. The present study aimed to address this gap by manipulating regulatory focus and measuring frontal cortical activity in 36 college students. Our results revealed that induced promotion focus led to increased left frontal cortical activity, whereas induced prevention focus led to increased right frontal cortical activity. These findings enhance our physiological understanding of regulatory focus and offer a deeper explanation of how regulatory focus influences alterations in psychology and behavior.

## 1. Introduction

Research suggests that the frontal cortex plays a crucial role in emotional and motivational processes [1,2]. Studies examining frontal cortical activity span various domains, including personality [3], clinical study [4], and social factors [5]. For instance, higher left frontal cortical activity is linked to greater positive affect [6], increased sensation-seeking [7], and heightened reward sensitivity [8]. In contrast, higher right frontal cortical activity is associated with greater negative affect [1], higher levels of depression and anxiety [4,9], greater social inhibition [10], and decreased risky decision making [11,12].

### 1.1. Two Main Models of Frontal Cortical Activity

Many conceptual perspectives have been proposed regarding the relationship between frontal cortical activity and emotional or motivational processes. One such perspective suggests a link between positive affect and increased left frontal cortical activity, and negative affect with increased right frontal cortical activity [13,14,15], known as the affective valence model. However, challenges to this model arise from studies finding that anger, a negative emotion with approach motivation, is associated with relatively greater left frontal cortical activity rather than greater right frontal cortical activity [16,17]. In response, researchers have proposed an alternative explanation, known as the motivational direction model [18]. This model suggests that approach motivation is associated with increased left frontal cortical activity, while avoidance motivation is associated with increased right frontal cortical activity [19]. This model suggests that approach motivation is associated with increased left frontal cortical activity, while avoidance motivation is associated with increased right frontal cortical activity [19]. Gray’s motivation theory posits that motivational direction is an organism’s tendency either to move towards (approach) goals (e.g., reward) or away from (avoidance) goals (e.g., punishment) [20,21,22]. This concept is instrumental in exploring the connection between approach–avoidance motivation and the frontal cortical activity. The left frontal cortex is implicated in approach behavior, functioning as a biological foundation for the pursuit of desired goals. It enables the representation of these goals, even without direct sensory cues, guiding behavior towards their attainment. On the flip side, the right frontal cortex is associated with avoidance or withdrawal behavior. It heightens anxious arousal and enhances visuospatial awareness of undesirable environmental stimuli, steering behavior away from such goals [23,24]. This delineation of roles between the left and right frontal cortices offers valuable insights into the neural underpinnings of motivational dynamics.

### 1.2. Behavioral Activation–Behavioral Inhibition Model and Frontal Cortical Activity

Despite the motivational direction model’s broad application across various fields in recent years, several studies have struggled to replicate findings concerning the line between approach/avoidance motivation and the specific activities of the left and right frontal cortices [10,25,26,27,28,29]. For example, some research indicates that avoidance motivation is not exclusively associated with right or left frontal cortical activity but rather with medial frontal cortical activity related to conflict processing (e.g., N2 and ERN ERP components) [30]. This is supported by findings from Amodio et al. (2007), which suggest that the approach–avoidance paradigm might not adequately explain certain responses, particularly in scenarios involving complex social emotions like guilt, which can trigger both behavioral inhibition and increased approach motivation [31]. To address these challenges, the behavioral activation–behavioral inhibition model of anterior asymmetry (BBMAA), inspired by the revised reinforcement sensitivity theory [22], has been proposed as a more fitting framework [10,32].

BBMAA has garnered significant attention and is widely supported as a more accurate model than the motivational direction model [33,34,35]. In contrast to the motivational direction model, BBMAA encompasses pure avoidance motivation mediated by the fight–flight–freezing system under behavioral activation [10,36]. This adjustment arises from the understanding that avoidance motivation can be transformed into approach motivation, such as during an escape from a predator, where avoidance motivation might transform into an approach toward safety. Essentially, behavioral activation can be instigated by either approach or avoidance motivation. In light of this, researchers position revised behavioral activation and behavioral inhibition as regulatory systems governing conflicts between approach and avoidance motivation from a biobehavioral perspective [12,28,37,38]. Furthermore, BBMAA correlates left frontal cortical activity with revised behavioral activation systems and right frontal cortical activity with revised behavioral inhibition (or goal conflict) systems. Revised behavioral activation and behavioral inhibition were assessed using imagery paradigms or self-report measures based on Gray’s updated theory of reinforcement sensitivity [22]. According to this updated theory, revised BAS is defined as goal-directed action, regardless of behavior direction, whereas the revised BIS concerns the inhibition of goal-directed actions triggered by goal conflicts. Previous studies have identified that goals stimulate the left-lateralized action planning system in the frontal cortex [10], potentially clarifying the link between the revised BAS and left frontal cortical activity. Additionally, research utilizing transcranial direct current stimulation (tDCS) indicates that the right frontal cortex plays a key role in managing goal conflict. Activating this region has been shown to mitigate motivationally conflicting behavior [39], offering insight into the connection between the revised BIS and right frontal cortical activity.

### 1.3. Regulatory Focus and Frontal Cortical Activity

Similar to the behavioral activation and inhibition systems, promotion and prevention focus represent higher-order motivational systems. They act as regulatory systems governing approach and avoidance motivations from a socio-cognitive perspective [38,40,41,42]. However, there has been relatively limited attention given to the relationship between promotion and prevention focus, and frontal cortical activity.

#### 1.3.1. Promotion and Prevention Focus

Regulatory focus theory distinguishes between two regulatory systems that are sensitive to different outcomes [43]. The promotion focus system, developed in response to the need for nurturance, is sensitive to positive outcomes [41,43]. Individuals in a promotion focus aim to maximize positive outcomes through eager means, translating into an inclination to say “yes” in a signal detection task (a risky bias) and ensuring “hits” (gains) [44,45]. The prevention focus system, developed in response to the need for security, is sensitive to negative outcomes [41,43]. Individuals in a prevention focus aim to minimize negative outcomes through cautious means, resulting in an inclination to say “no” in a signal detection task (a conservative bias) and ensuring “correct rejections” (non-losses) [44,45]. Additionally, some research has revealed that promotion-focused individuals tend towards faster but less accurate responses in a signal detection task, while those in a prevention focus exhibit the opposite pattern [46,47].

#### 1.3.2. Promotion and Prevention Focus and Frontal Cortical Activity

While some fMRI studies have recognized the link between regulatory focus and frontal cortical activation [48,49,50,51], empirical exploration of this relationship through resting electroencephalogram (EEG) data remains scarce. To date, only one EEG study has identified a correlation where a promotion focus is linked to increased left frontal cortical activity and a prevention focus to enhanced right frontal cortical activity [52]. Although fMRI provides superior spatial resolution for delineating brain activity across different regions, it is hampered by its relatively poor temporal resolution, which fails to capture the swift fluctuations in brain dynamics. In contrast, EEG excels in directly measuring neuronal electrical activity, offering immediate insights into neural processes that fMRI, which measures physiological changes like blood oxygenation and flow, cannot. The reliance solely on fMRI results without integrating EEG data may restrict a comprehensive understanding of brain functions. Moreover, prior investigations have largely concentrated on correlation analysis without delving into causality. This absence of causative analysis curtails the predictive capacity of studies, challenging the ability to anticipate how shifts in regulatory focus might influence frontal cortical activity over time.

### 1.4. The Present Research

The current study seeks to investigate the influence of regulatory focus on frontal cortical activity, aiming to provide causal evidence for the association between promotion/prevention focus and left/right frontal cortical activity. Drawing upon previous research, the current study hypothesized that induced promotion focus would lead to greater left frontal cortical activity, while induced prevention focus would lead to greater right frontal cortical activity. To test these hypotheses, an experiment was designed wherein participants were randomly assigned to two groups, undergoing recording of behavioral responses, measurement of state regulatory focus, and collection of resting-state EEG data.

## 2. Materials and Methods

### 2.1. Participants

Thirty-six college students (30 females; aged 20.47 ± 2.17 years) were recruited from a university in China as paid participants. They were randomly assigned to either the prevention or promotion focus manipulation groups, with 18 participants in each group (see Table 1). This study did not exclude any participants. Each participant completed the tasks diligently without withdrawing midway. Right-handed individuals were selected to minimize potential physiological variations associated with frontal cortical activity. All participants were unmarried; had a normal or corrected-to-normal vision; and reported no history of psychiatric disorders, neurological issues, or brain trauma. Prior to the commencement of the experiment, participants provided their handwritten informed consent. The current study was reviewed and approved by the Institutional Review Board at the corresponding author’s institution before being conducted (consent number: BNU202212080126).

### 2.2. Self-Report Measures

Chronic regulatory focus was measured using 18 items adapted from the work of Lockwood et al. [53]. This included 9 items for promotion focus (α = 0.88), such as “Recently, I am focused on achieving positive outcomes”, and 9 items for prevention focus (α = 0.86), such as “Recently, I am focused on preventing negative events”. All items were rated using a seven-point scale (1 = strongly disagree, 7 = strongly agree).

Positive and negative emotions were measured using the general version of the Positive Affect (PA; α = 0.88) and Negative Affect (NA; α = 0.89) scales (PANAS, [54]) to assess participants’ current emotional state. The PANAS consists of 10 positive adjectives (such as proud and excited) and 10 negative adjectives (such as distressed and anxious). Participants rated their responses on a seven-point Likert scale (1 = not at all, 7 = extremely).

### 2.3. Procedure

Before the commencement of the study, participants were informed that they needed to complete questionnaires assessing their chronic regulatory focus and current emotional state. Following these measurements, participants underwent EEG recording, during which 8 min of baseline resting EEG data were acquired. Subsequently, participants needed to complete two tasks: a word categorization task and a recognition task, without explicit disclosure of the experimental aims. In reality, these two successive tasks aimed to induce regulatory focus, and both tasks were administered to ensure a robust manipulation [55]. All participants were randomly assigned to either the promotion focus manipulation group, or the prevention focus manipulation group.

In the word categorization task (adapted from the work of Lockwood et al. [53]), participants received a list of 24 words and were instructed to sort them into two categories. Participants created their categories, as labels for the two groups were not provided. Twelve words were related to cooking (e.g., pot, spoon), while the remaining 12 words were related to promotion focus (e.g., gain, win) in the promotion focus manipulation group or prevention focus (e.g., punishment, mistake) in the prevention focus manipulation group. No time limit was imposed for the categorization task.

Following the word categorization task, participants engaged in a recognition task, which required them to identify old words that appeared in the prior word categorization task. For the recognition task, participants viewed a set of 20 words and judged whether they had seen them before. Among these 20 words, 10 words were target items from the original list, including 5 old words related to cooking and 5 old words related to promotion/prevention focus. The remaining 10 words, not from the original list, were used as new distractor items, comprising 5 new words related to cooking and 5 words related to promotion/prevention focus.

In the recognition task, promotion and prevention focus manipulation were also embedded in the task instructions (adapted from the work of Higgins et al. [56]). Participants in the promotion focus manipulation group were informed, “The initial payment for this task is 20 yuan. If your performance exceeds 60% of the pre-test group, you will earn an additional 10 yuan. However, if you do not perform above 60% of the pre-test group, no additional money will be received”. Participants in the prevention focus manipulation group were informed “The initial payment for this task is 30 yuan. If your performance falls below 60% of the pre-test group, you will lose 10 yuan. However, if you perform at least 60% of the pre-test group, no money will be lost”.

After completing the recognition task, participants rated their state regulatory focus using four items adapted from Lockwood et al. [53] (e.g., “In the task just now, I was more focused on not making mistakes”). Following the completion of the four items, participants were notified about the need for additional resting EEG data collection. At this stage, 2 min of resting EEG data were acquired, with each open and closed eye period lasting for 1 min.

### 2.4. EEG Recording and Preprocessing

To obtain baseline measures of the EEG, participants were instructed to relax with their eyes open or closed. This followed one of two alternating orders (i.e., C, O, O, C, O, C, C, O, or O, C, C, O, C, O, O, C) with 1 min intervals for a total of 8 min, as in previous research [57]. EEG was also recorded for 2 min after the regulatory focus manipulation. Participants were instructed to keep their head and body as still as possible during the recordings, and instructions were provided via an audio embed with E-Prime. For EEG recording, a 32 channel electrode cap with the NeuroScan acquisition system (Herndon, VA, USA) using the 10–20 international system [58] was employed. The reference electrode was placed on the left ear lobe (A1), and data were also acquired from an electrode placed on the right ear lobe (A2). All impedances were kept under 5 KOhms, and the sample rate was 1000 Hz.

Offline data analysis was conducted using MATLAB R2022b with the EEGLAB v2020 toolbox [59]. A band-pass filter within the range of 0.1 to 40 Hz isolated relevant frequency components, and data were re-referenced to the mastoid average (A1/2). The continuous 8 min resting-state recording during the baseline period and the continuous 2 min resting-state recording during the post-manipulation period were segmented into 2 s epochs with a 1 s overlap. Moreover, independent component analysis (ICA) [59,60] was applied to remove components associated with eye movements, muscle-related artifacts, heartbeats, and line noise. Finally, each participant had a minimum of 10 s of artifact-free EEG data during each period (baseline and post-manipulation). This criterion ensured that an adequate amount of EEG data was available to compute stable estimates of alpha power [61].

### 2.5. Spectral Analysis

All artifact-free epochs were extracted using a Hanning window. Contiguous epochs were overlapped by 50% to minimize data loss during Hanning window extraction. Power spectra were calculated via a fast Fourier transform from F3 and F4, following the standard in frontal cortical activity research [62,63]. These power values were averaged across 2 s epochs, and total power within the alpha (8–13 Hz) frequency range was obtained. The data were natural log (ln) transformed into normalized distributions. As alpha power is inversely related to cortical activity, lower scores on the F3 indices indicated greater left frontal cortical activity, while lower scores on the F4 indices indicated greater right frontal cortical activity.

## 3. Results

### 3.1. Manipulation Check: Self-Report Results

**Figure 1:**
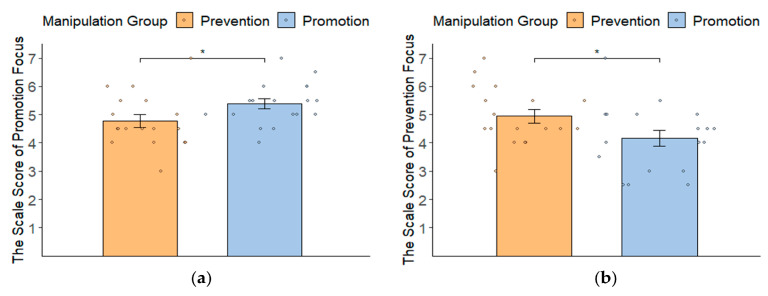
The scale score (mean ± SD) of state promotion focus (**a**) and prevention focus (**b**) of two groups (promotion vs. prevention focus manipulation group) after manipulation tasks. * *p* < 0.05. Error bars represent ± SE.

To test the hypothesis that induced promotion focus increases left frontal cortical activity and induced prevention focus increases right frontal cortical activity, we first needed to determine the effectiveness of the regulatory focus manipulation. A multivariate analysis of variance (MANOVA) was conducted with the manipulation conditions as the independent variable and the two regulatory focus scale as the dependent variables (see Figure 1). The results showed that participants in the promotion focus manipulation group reported stronger promotion focus (*M* = 5.39 ± 0.74) than participants in the prevention focus manipulation group (*M* = 4.78 ± 0.94, *F*(1, 34) = 4.69, *p* = 0.038, $\eta_{p}^{2}$ = 0.12) (see Figure 1a). Conversely, participants in the prevention focus manipulation group reported a stronger prevention focus (*M* = 4.94 ± 1.01) than participants in the promotion focus manipulation group (*M* = 4.17 ± 1.20; *F*(1, 34) = 4.13, *p* = 0.043, $\eta_{p}^{2}$ = 0.12) (see Figure 1b). These results demonstrated that the manipulation was effective.

### 3.2. Manipulation Check: Behavioral Results

**Figure 2:**
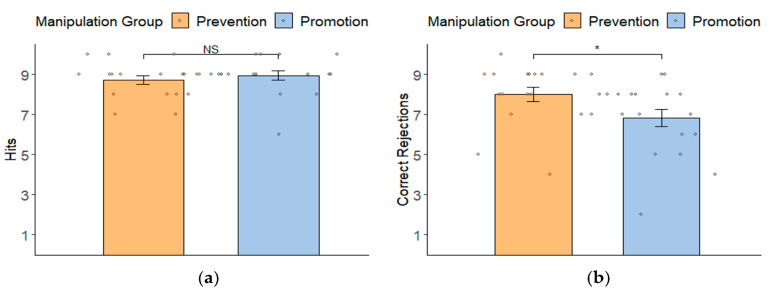
Response bias (mean ± SD) regarding hits (**a**) and correct rejections (**b**) of two groups (promotion vs. prevention focus manipulation group) in the recognition task. * *p* < 0.05; NS = not significant; error bars represent ± SE.

Response bias. A MANOVA was conducted to analyze two types of response bias (hits and correct rejections) with a between-factor (group: promotion vs. prevention focus manipulation group) (see Figure 2). For hits (see Figure 2a), the difference between two manipulation groups was not significant (*M_promotion focus manipulation group_* = 8.94 ± 0.94 vs. *M_prevention focus manipulation group_* = 8.72 ± 0.89; *F*(l, 34) = 0.53, *p* = 0.472, $\eta_{p}^{2}$ = 0.02). For correct rejections (see Figure 2b), there was a significant difference between the two groups (*F*(1, 34) = 4.23, *p* = 0.047, $\eta_{p}^{2}$ = 0.11). Participants in the prevention focus manipulation group made more correct rejections (*M* = 8.00 ± 1.53) than participants in the promotion focus manipulation group (*M* = 6.83 ± 1.86). The results demonstrated that prevention focus led individuals to be more inclined to avoid making mistakes.

**Figure 3:**
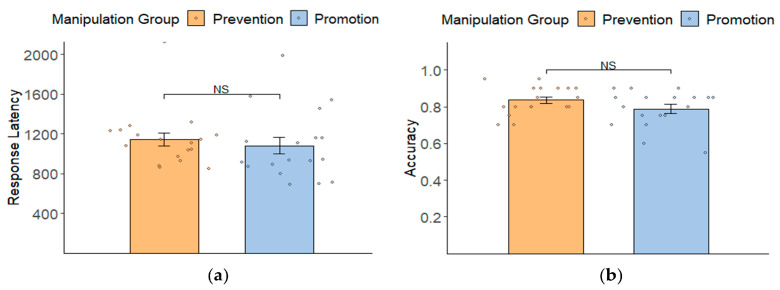
Response latency (**a**) and accuracy (**b**) (mean ± SD) of two groups (promotion vs. prevention focus manipulation group) in the recognition task. NS = not significant. Error bars represent ± SE.

Response latency and accuracy. Two independent-sample T-tests were conducted to analyze response latency and accuracy, separately. The results showed that there was no significant difference in response tendency (*M_promotion focus manipulation group_* = 1082.20 ± 353.59 ms vs. *M_prevention focus manipulation group_* = 1144.78 ± 292.87 ms; *t*(34) = -0.57, *p* = 0.574, Cohen’s d = 0.19) (see Figure 3a). Additionally, there was also no significant difference in accuracy (*M_promotion focus manipulation group_* = 0.79 ± 0.10 vs. *M_prevention focus manipulation group_* = *M* = 0.84 ± 0.08; *t*(34) = −1.58, *p* = 0.122, Cohen’s d = 0.53) (see Figure 3b).

### 3.3. EEG Results

**Figure 4:**
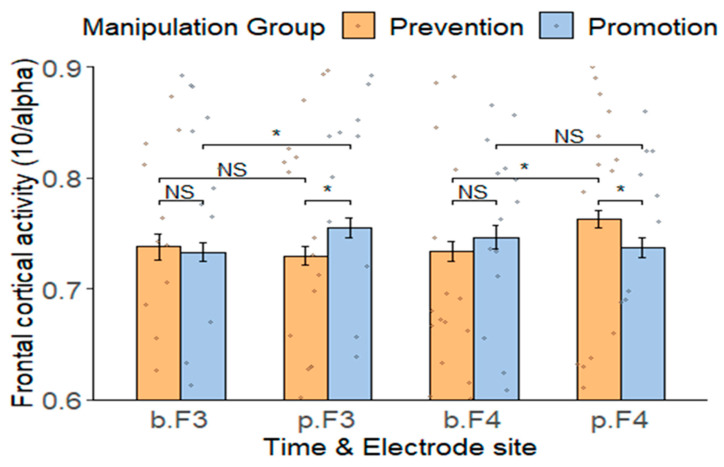
Frontal cortical activity (mean ± SD) at F3 and F4 electrode sites in two groups (promotion vs. prevention focus manipulation group) during two time periods (baseline vs. post-manipulation). Frontal cortical activity is obtained by dividing 10 by the alpha power. b = baseline period; p = post-manipulation period. * *p* < 0.05; NS = not significant. Error bars represent ± SE.

Our primary hypothesis posited that the regulatory focus manipulation would influence left or right frontal cortical activity. A 2 × 2 × 2 repeated-measures analysis of variance was conducted to analyze alpha power with a between-factor (group: promotion vs. prevention focus manipulation group) and two within-factors (time: baseline vs. post-manipulation period; electrode site: F3 vs. F4) (see Figure 4). The results showed a significant three-way interaction (*F(1, 34)* = 17.71, *p* < 0.001, $\eta_{p}^{2}$ = 0.34). To break down the three-way interaction, we tested the time by group interaction for the F3 and F4 electrode sites. For the left frontal cortical activity (obtained from the F3 electrode site), there was a significant time by group interaction (*F(1, 34)* = 5.13, *p* = 0.030, $\eta_{p}^{2}$ = 0.13). Simple effect analysis found that during the post-manipulation period, higher left frontal cortical activity was induced by the promotion focus manipulation group (*M* = 13.28 ± 0.66) compared to the prevention focus manipulation group (*M* = 13.74 ± 0.67; *F(1, 34)* = 4.28, *p* = 0.046,$\;\eta_{p}^{2}$ = 0.11), with no significant difference in left frontal cortical activity between the two groups during the baseline period (*M_promotion focus manipulation group_* = 13.67 ± 0.67 vs. *M_prevention focus manipulation group_* = 13.61 ± 0.93, *F(1, 34)* = 0.05, *p* = 0.825, $\eta_{p}^{2}$ = 0.001). Additionally, for the promotion focus manipulation group, left frontal cortical activity induced during the post-manipulation period (*M* = 13.28 ± 0.66) was higher than during the baseline period (*M* = 13.67 ± 0.67; *F(1, 34)* = 5.97, *p* = 0.020, $\eta_{p}^{2}$ = 0.15), whereas for the prevention focus manipulation group, there was no significant difference in left frontal cortical activity between the post-manipulation period (*M* = 13.74 ± 0.67) and the baseline period (*M* = 13.61 ± 0.93, *F(1, 34)* = 0.58, *p* = 0.452, $\eta_{p}^{2}$ = 0.02).

For right frontal cortical activity (obtained from the F4 electrode site), there was a significant time by group interaction (*F(1, 34)* = 7.64, *p* = 0.009, $\eta_{p}^{2}$ = 0.18). Simple effect analysis found that during the post-manipulation period, higher right frontal cortical activity was induced by the prevention focus manipulation group (*M* = 13.13 ± 0.59) compared to the promotion focus manipulation group (*M* = 13.59 ± 0.69; *F(1, 34)* = 4.72, *p* = 0.037 $\eta_{p}^{2}$ = 0.12), with no significant difference in right frontal cortical activity between the two groups during the baseline period (*M_promotion focus manipulation group_* = 13.44 ± 0.82 vs. *M_prevention focus manipulation group_* = 13.66 ± 0.72, *F(1, 34)* = 0.68, *p* = 0.414, $\eta_{p}^{2}$ = 0.02). Additionally, for the prevention focus manipulation group, right frontal cortical activity induced during the post-manipulation period (*M* = 13.14 ± 0.59) was higher than during the baseline period (*M* = 13.66 ± 0.72; *F(1, 34)* = 9.23, *p* = 0.005, $\eta_{p}^{2}$ = 0.21); however, for the promotion focus manipulation group, there was no significant difference in right frontal cortical activity between the post-manipulation period (*M* = 13.59 ± 0.69) and the baseline period (*M =* 13.44 ± 0.82, *F(1, 34)* = 0.76, *p* = 0.390, $\eta_{p}^{2}$ = 0.02). These results supported our hypothesis.

## 4. Discussion

There is currently a limited body of neuroscience research on the relationship between regulatory focus and frontal cortical activity, despite the clear importance of understanding how individuals initiate behavior to achieve personal goals in various aspects of human behavior. Previous research has primarily focused on conceptual derivation and correlation analyses, lacking concrete evidence of causality. Our results reveal that induced promotion focus increased left frontal cortical activity, while induced prevention focus increased right frontal cortical activity. Additionally, as predicted, participants in the prevention focus manipulation group made more correct rejections than participants in the promotion focus manipulation group. The current study provides causal evidence for the relationship between regulatory focus and frontal cortical activity. Furthermore, the current study expands the correlation between regulatory systems and frontal cortical activity from a biobehavioral perspective to a socio-cognitive perspective, improving our comprehension of the frontal cortex’s role in goal attainment.

What differs from previous research is that the result was observed in the mid-frontal cortex (F3/4; mainly dorsolateral prefrontal cortex [11,64]), with no corresponding activity detected in the lateral frontal cortex (F7/8). This discrepancy may be attributed to the mid-frontal cortex’s close association with regulatory focus. Packer and Cunningham employed fMRI and identified heightened activity in the mid-frontal cortex associated with promotion goals [51]; Johnson et al.’s work found that activity in the medial frontal cortex reflected acute regulatory focus states [50]. These findings align with our current results. Although we observed similar activity patterns in the F7 and C3 sites as in the F3 sites (see Supplementary Materials, Table S1), it is conceivable that the activation of alpha rhythms over the mid-frontal regions stimulated ipsilateral lateral frontal and central regions via cortico-cortical connections [65]. Synthesizing both neuroanatomical and functional imaging studies, our findings support the specificity of the mid-frontal cortex in regulatory focus.

### 4.1. Regulatory Focus and Approach and Avoidance Motivation

Our study enriches the understanding of how regulatory focus influences approach and avoidance motivations. We discovered that a promotion focus increases activity in the left frontal cortex, while a prevention focus boosts activity in the right frontal cortex. This finding is consistent with prior studies that have linked approach and avoidance motivations to activity in the left and right frontal cortices, respectively, highlighting the intricate link between regulatory focus and motivational dynamics [66]. For instance, a promotion focus is associated with behaviors geared towards approaching goals, whereas a prevention focus tends to predict behaviors aimed at avoiding goals [56,67]. The research by Higgins et al. illustrated this by showing that individuals with a promotion focus are more likely to recall life events where they sought to approach desirable states (e.g., supporting a friend), while those with a prevention focus remembered more instances of avoiding undesirable outcomes (e.g., not losing touch with a friend) [68]. This pattern suggests a parallel between the impact of regulatory focus and the classical approach–avoidance motivation on frontal cortical activity.

However, while the parallels between promotion focus with approach motivation and prevention focus with avoidance motivation are evident, it is critical to delineate the nuances between regulatory focus and these motivations. Regulatory focus pertains to how people use approach and avoidance strategies to fulfill their goals, indicating a layer of strategic regulation over basic motivational directions. Previous studies distinguish approach and avoidance motivations as the innate impulses to move “toward” or “away from” specific outcomes [22,66]. Conversely, both promotion and prevention focuses are strategies that involve moving towards desirable outcomes or away from undesirable ones, but with distinct emphases on achieving gains or avoiding losses [41]. Understanding these nuances sheds light on the importance of looking beyond mere approach and avoidance motivations when studying regulatory focus, offering a more nuanced view of how individuals pursue their goals.

### 4.2. Regulatory Focus and Frontal Cortical Activity

Our findings contribute to enhancing the comprehension of the consequences of regulatory focus from an electrophysiological perspective. Research on regulatory focus has primarily concentrated on its psychological and behavioral impacts. The current findings explain the underlying physiological mechanisms through which regulatory focus influences individual behavior. For instance, the current study revealed that individuals in a promotion focus tend to display risky bias, as indicated by more hits and shorter response latency. Conversely, individuals in a prevention focus tend to display conservative bias, as indicated by more correct rejections and higher accuracy. These findings (although some differences are not significant) are consistent with previous research [43,56]. Traditional interpretations of these findings have primarily been rooted in the psychological level, suggesting that individuals in a promotion focus prioritize nurturant needs, resulting in risky bias. Conversely, individuals in a prevention focus prioritize safety needs, leading to conservative bias [44,69]. However, our study could offer an alternative explanation of this phenomenon from a physiological level. Specifically, individuals in a promotion focus may exhibit increased left frontal cortical activity, which is linked to sensation-seeking and impulsive behavior [7,25]. As a result, individuals in a promotion focus may display a tendency towards risk-taking behaviors. Conversely, individuals in a prevention focus may exhibit increased right frontal cortical activity, which is associated with fear of memories and loss aversion [12,70]. Consequently, individuals in a prevention focus may lean towards conservative behavior. Our findings contribute to a deeper understanding of the consequences of regulatory focus by providing a physiological explanation, thereby enriching our comprehension of social phenomena from a neurobiological perspective.

### 4.3. Limitations

On the one hand, regarding behavioral outcomes, unfortunately, we only observed the expected trends in some variables of interest, such as hits, response latency, and accuracy. These insignificant results in behavior were common in previous research, as evidenced by studies indicating no significant main effect of regulatory focus on response latency and recognition accuracy [44,71]. Several factors contributed to these insignificant results. Firstly, it may be due to the experimental design being too simplistic. Although there was no explicit hint for participants to memorize words in the word categorization task, we did not impose restrictions on the categorization time. The fewer number of words needed recognition and unrestricted categorization time may contributed to higher hits, shorter response latency, and higher accuracy among participants. Secondly, this may be attributed to the sensitivity of behavioral response [72]. Relative to high temporal resolution EEG recording, the behavioral response may be influenced by additional mental and cognitive mechanisms, such as individuals’ perceived difficulty in tasks [73] and individuals’ cognitive ability to suppress unrelated information [74], leading to delays and reduced sensitivity. Lastly, the small sample size might be another reason for the lack of significance in the observed differences. Therefore, future investigations could advance by employing more sophisticated experimental designs, utilizing stringent metrics, and examining larger sample populations to strengthen the validity of these findings. On the other hand, while we highlighted the fundamental differences and close connections of regulatory focus with approach and avoidance motivation, there has been no study simultaneously comparing regulatory focus with approach and avoidance motivation regarding their impact on frontal cortical activity. Future research could, through meticulous design, compare the influence of both on frontal cortical activity, shedding light on the intricate interplay between motivational processes and neural mechanisms underlying goal pursuit and decision making. Additionally, such investigations could offer valuable insights into potential therapeutic interventions targeting regulatory processes for various cognitive and affective disorders.

## 5. Conclusions

Our study is the first to investigate the causal relationship between regulatory focus and frontal cortical activity, providing socio-cognitive evidence concerning the association between regulatory systems and frontal cortical activity. Specifically, our findings demonstrate that induced promotion focus increases left frontal cortical activity, while induced prevention focus increases right frontal cortical activity.

## Figures and Tables

**Table 1:** Demographical characteristics of promotions and prevention focus manipulation groups (mean ± SD).

Items	Promotion Focus Manipulation Group (N = 18)	Prevention Focus Manipulation Group (N = 18)	t	*p*
Chronic prevention focus	5.07 ± 0.98	4.55 ± 0.93	1.63	0.11
Chronic promotion focus	5.05 ± 0.87	4.98 ± 0.75	0.25	0.80
Positive emotion	4.02 ± 0.89	4.34 ± 0.74	−1.14	0.26
Negative emotion	2.41 ± 1.01	2.11 ± 0.87	0.95	0.35
Socioeconomic status	4.56 ± 1.04	5.06 ± 1.39	−1.22	0.23
Age (years)	20.39 ± 2.06	20.56 ± 2.33	−0.23	0.82
Gender (male/female)	2/16	4/14	0.88	0.39

Independent samples *t*-test was performed (two-tailed).

## Data Availability

The data presented in this study are available on request from the first author.

## Supplementary Materials

The following supporting information can be downloaded at https://www.mdpi.com/article/10.3390/bs14040292/s1. Table S1. Alpha power scores at electrode sites (mean ± SD).
