# Supplementary material for: The Effect of Induced Regulatory Focus on Frontal Cortical Activity
Source: Behav Sci (Basel). 2024 Apr 1;14(4):292. doi: 10.3390/bs14040292 (PMC11047718; doi:10.3390/bs14040292)
Supplement: Supplementary file 1 [file behavsci-14-00292-s001.zip › behavsci-2915645-supplementary.pdf]

Table S1. Alpha power scores at electrode sites(Mean ± SD）

Electrode sites	Time & Manipulation Group				F test
	Baseline & Promotion	Baseline &Prevention	Post-M & Promotion	Post-M & Prevention	
F7	13.84 ±0.83	13.51 ±1.13	13.65±0.61	14.05±0.82	F (1,34) =4.41, p=0.04, η_p^2=0.12
F8	13.58±1.09	13.71±1.12	13.60±0.74	13.58±0.66	F (1,34) =1.57, p=0.70, η_p^2=0.01
C3	14.18±0.74	13.93±0.91	13.77±0.80	14.14±0.74	F (1,34) =6.84, p=0.01, η_p^2=0.17
C4	14.01±0.88	14.09±0.90	14.04±0.72	13.72±0.79	F (1,34) =2.19, p=0.15, η_p^2=0.06
T3	13.61±0.68	13.51±0.97	13.38±0.79	13.85±0.63	F (1,34) =3.73, p=0.07, η_p^2=0.09
T4	13.62±0.83	13.71±0.70	13.74±0.72	13.5±0.68	F (1,34) =1.67, p=0.20, η_p^2=0.05
P3	14.53±1.23	14.34±0.90	14.71±1.00	14.64±0.92	F (1,34) =0.12, p=0.73, η_p^2=0.00
P4	14.72±1.00	14.65±1.00	14.83±0.84	14.21±1.15	F (1,34) =2.86, p=0.10, η_p^2=0.08
O1	14.79±1.16	14.46±1.11	14.83±1.31	14.56±1.24	F (1,34) =0.17, p=0.90, η_p^2=0.00
O2	15.29±1.05	14.53±1.00	15.07±1.34	14.49±1.16	F (1,34) =0.29, p=0.59, η_p^2=0.01

Note. The results of the 2×2 analysis of variance (ANOVA) for the 10 electrodes of interest in previous research, with a between-factor (group: promotion vs. prevention focus manipulation group) and a within-factor (time: baseline vs. post-manipulation). Alpha power is inversely related to cortical activity, so lower scores reflect greater cortical activity. Baseline & Promotion, the baseline period & the promotion focus manipulation group; Baseline & Prevention, the baseline period & the promotion focus manipulation group; Post-M & Promotion, the post-manipulation period & the promotion focus manipulation group; Post-M & Prevention, the post-manipulation period & the prevention focus manipulation group.
